# Six-month follow-up of multidomain cognitive impairment in non-hospitalized individuals with post-COVID-19 syndrome

**DOI:** 10.1007/s00406-024-01863-3

**Published:** 2024-07-24

**Authors:** Ann-Katrin Schild, Daniel Scharfenberg, Anton Regorius, Kim Klein, Lukas Kirchner, Goereci Yasemin, Joachim Lülling, Dix Meiberth, Finja Schweitzer, Gereon R. Fink, Frank Jessen, Christiana Franke, Oezguer A. Onur, Stefanie Theresa Jost, Clemens Warnke, Franziska Maier

**Affiliations:** 1 Department of Psychiatry, Faculty of Medicine and University Hospital Cologne, University of Cologne, Cologne, Germany; 2 Department of Medical Psychology ǀ Neuropsychology and Gender Studies and Center for Neuropsychological Diagnostics and Intervention (CeNDI), Faculty of Medicine and University Hospital Cologne, University of Cologne, Cologne, Germany; 3 Department of Psychology, Clinical Psychology, Experimental Psychopathology, and Psychotherapy, Philipps University Marburg, Marburg, Germany; 4 Department of Neurology, Faculty of Medicine and University Hospital Cologne, University of Cologne, Cologne, Germany; 5 Department of Neurology with Experimental Neurology, Charité-Universitätsmedizin Berlin, Corporate Member of Freie Universität Berlin, Humboldt-Universität zu Berlin, Berlin Institute of Health, Berlin, Germany; 6 Cognitive Neuroscience, Institute of Neuroscience and Medicine (INM-3), Research Centre Jülich, Jülich, Germany; 7 German Center for Neurodegenerative Diseases (DZNE), Bonn, Germany; 8 Excellence Cluster on Cellular Stress Responses in Aging-Associated Diseases (CECAD), University of Cologne, Cologne, Germany

**Keywords:** Neuropsychology, Long COVID, Neurocognitive disorder, Cognitive deficits, Subjective complaints, SARS-CoV-2

## Abstract

Some people infected with SARS-CoV-2 report persisting symptoms following acute infection. If these persist for over three months, they are classified as post-COVID-19 syndrome (PCS). Although PCS is frequently reported, detailed longitudinal neuropsychological characterization remains scarce. We aimed to describe the trajectory of cognitive and neuropsychiatric PCS symptoms. 42 individuals with persisting cognitive deficits after asymptomatic to mild/moderate acute COVID-19 at study inclusion received neuropsychological assessment at baseline (BL) and follow-up (FU; six months after BL). Assessments included comprehensive testing of five neurocognitive domains, two cognitive screening tests, and questionnaires on depression, anxiety, sleep, fatigue, and health-related quality of life. Results showed high rates of subjective cognitive complaints at BL and FU (95.2% versus 88.1%) without significant change over time. However, objectively measured neurocognitive disorder (NCD) decreased (61.9% versus 42.9%). All cognitive domains were affected, yet most deficits were found in learning and memory, followed by executive functions, complex attention, language, and perceptual motor functions. In individuals with NCD, the first three domains mentioned improved significantly over time, while the last two domains remained unchanged. Cognitive screening tests did not prove valuable in detecting impairment. Neuropsychiatric symptoms remained constant except for quality of life, which improved. This study emphasizes the importance of comprehensive neuropsychological assessment in longitudinal research and provides valuable insights into the trajectory of long-term neuropsychological impairments in PCS. While cognitive performance significantly improved in many domains, neuropsychiatric symptoms remained unchanged.

**Supplementary Information:**

The online version contains supplementary material available at 10.1007/s00406-024-01863-3.

## Introduction

There is growing evidence that symptoms occurring during the acute phase of severe acute respiratory syndrome coronavirus 2 (SARS-CoV-2) infection persist or that even new symptoms can appear after the acute phase [1, 2]. Various terms describe this condition: Long-COVID refers to symptoms that persist for at least four weeks after infection. Post-COVID-19 syndrome (PCS) is used if symptoms develop during or after COVID-19, continue for more than 12 weeks after infection, and cannot be explained by an alternative diagnosis [3]. The definition of the post-COVID-19 condition published by the World Health Organisation (WHO) adds symptoms that persist for at least two months and impact everyday functioning [4].

A recent umbrella review regarding Long-COVID showed prevalence estimates ranging from 7.5 to 41% [5]. Estimates suggest that 65 million individuals worldwide may suffer from Long-COVID [6]. Focussing explicitly on PCS, Fernandez-de-las-Peñas et al. reported that 45.9% of individuals exhibited at least one post-COVID symptom [7]. Altogether, these studies indicate that long-term symptoms following COVID-19 are frequent.

Frequent PCS symptoms include neuropsychiatric symptoms, such as fatigue and affective symptoms. Based on data from a meta-analysis, the estimated prevalence of fatigue in PCS is 37% [8]. Prevalence rates for affective symptoms such as depression and anxiety are estimated to range from 12 to 23% [8, 9]. Additionally, neuropsychiatric PCS symptoms may also manifest as sleep disturbance (27.4%) or objective cognitive impairment (20.2%) [1].

A meta-analysis that focused on cognitive impairment reported brain fog (32%), memory issues (27%), and attention disorder (22%) as common PCS symptoms [8]. Further, a review of objective test data on cognitive impairment found deficits in global cognitive function in all 12 studies, ranging from 15 to 80% in the samples [10]. In our cohort of individuals with PCS with subjective cognitive complaints, objective cognitive impairment was found in about 60% of individuals when applying extensive neuropsychological testing and was prevalent across all cognitive domains assessed (the focus of impairments were domains of learning and memory, attention, and executive functioning) [11]. These findings were underlined by a review of objective test data that found impairments in attention and executive functions, memory, and visuospatial cognition [10]. Cognitive impairments impact life severely and are associated with lower quality of life and higher impairment at work [12].

Disease severity of acute COVID-19 (i.e., hospitalization, number of initial symptoms, and intensive care unit admission) has been identified as one risk factor for PCS [13–15]. However, persistent symptoms are common even in individuals with asymptomatic, mild, or moderate (i.e., non-hospitalized) acute COVID-19, although prevalence estimates vary considerably [5, 16]. Considering recent findings, showing that 92.4% of individuals diagnosed with COVID-19 did not require hospitalization [17], the absolute number of individuals at risk of PCS is still highly relevant.

Studies investigating the prevalence of cognitive impairment in hospitalized versus non-hospitalized individuals as a PCS symptom could not detect differences between the two groups [18] or found even higher estimates for cognitive impairment in non-hospitalized individuals [8]. This observation suggests that cognitive impairment is at least as frequent in non-hospitalized as it is within in hospitalized individuals, an implication that is supported by other studies [19–22].

It is highly relevant for clinicians and scientists whether cognitive impairment is reversible or not as in neurodegenerative diseases [23]. Post-infectious syndromes have been known since the Spanish influenza in 1918 and can help deducing possible long-term consequences of COVID-19 [24, 25]. A review published before the COVID-19 pandemic, describes that when an infection caused acute respiratory distress syndrome (ARDS) 46–80% of patients experienced cognitive impairment one year after hospital discharge, five years later 20% of patients reported cognitive impairment [26]. The most affected domains were executive functions, memory, concentration, and attention [27]. Mild as well as severe influenza is associated with cognitive impairment and an increased long-term risk for neurodegenerative conditions [23, 25].

As is known from the other coronaviruses severe acute respiratory syndrome (SARS) and Middle East respiratory syndrome (MERS) long-term cognitive impairment is possible after the acute phase of the disease with deficits in concentration and attention, as well as in memory lasting up to several months [28]. Little is known about the trajectory of neuropsychological and neuropsychiatric symptoms in PCS. A systematic review of hospitalized and non-hospitalized individuals examined different neuropsychiatric symptoms like cognitive deficits, fatigue, anxiety, depression, post-traumatic stress disorder, and sleep disturbances for up to seven months [29]. Symptoms were assessed at varying time points and indicated a trend toward improvement, even though primary studies yielded mixed results. In addition, meta-analyses indicated no significant differences in the prevalence of cognitive deficits and fatigue symptoms between mid-term (i.e., 3–6 months after infection) and long-term follow-up (i.e., at least six months after infection) [8, 18]. However, symptoms of anxiety and depression increased from mid-term to long-term follow-up [8].

Different primary studies that examined the trajectory of neuropsychiatric symptoms from acute COVID-19 up to 12 months after diagnosis using online surveys showed an increase in cognitive and neuropsychiatric symptoms (e.g., memory problems, confusion, fatigue, anxiety, depression), particularly in the early period after acute COVID-19, followed by either a decrease or stagnation [30, 31]. In a study applying extensive neuropsychological testing to 128 individuals at three time points (two, four, twelve months), cognitive performance was significantly different compared to normative reference at any time with mild to moderate cognitive impairment ranging from 16 to 26%. Cognition declined over time although the proportion of cognitive impairment did not differ significantly [32].

In summary, the current literature does not provide consistent findings on the trajectory of cognitive and neuropsychiatric symptoms over time. Moreover, various factors of the studies conducted render their comparability difficult, such as different methods of assessment (subjective information versus objective testing), the use of comprehensive neuropsychological tests versus cognitive screening tests, in-person versus remote testing, different follow-up periods, and heterogeneous samples (e.g., age, severity of COVID-19, sex, education, concomitant medication, pre-existing co-morbidities). However, analyzing intraindividual trajectories of cognitive and neuropsychiatric symptoms of individuals with PCS over time can be of great importance for improving therapeutic decisions. Results could help to identify symptoms that seem to remit on their own, while others might need to be specifically targeted by neuropsychological or psychotherapeutic interventions.

Our study constitutes the longitudinal follow-up of earlier work [11] that aimed to include comprehensive in-person neuropsychological assessments of five neurocognitive domains defined by the Diagnostic and Statistical Manual [33]. Here, we included individuals who reported subjective cognitive complaints after mild to moderate SARS-CoV-2 infection. The main goal of the current study was to examine whether the extent of cognitive deficits changes over time. Our extensive neuropsychological test battery allowed us to focus on domain-specific changes and global cognition. Additionally, we analyzed neuropsychiatric symptoms and health-related quality of life over time.

## Participants and methods

A prospective cohort of non-hospitalized individuals with PCS was recruited at the University Hospital of Cologne. Participants were seeking medical advice due to persistent symptoms following COVID-19 and either contacted us directly or were referred by the Department of Infectious Diseases. The Institutional Review Board granted ethical approval (20–1501). The study was registered in the German Clinical Trials Register (DRKS00024434). Participants were individuals reporting subjective cognitive complaints or fatigue at study inclusion at least three months after mild to moderate confirmed COVID-19. Known premorbid mild cognitive impairment, dementia, or a history of severe psychiatric or neurological conditions within the last two years were defined as exclusion criteria. For further information on the participant recruitment process and inclusion/exclusion criteria, see Schild et al. [11]. Baseline (BL) assessment (see Online Resource Table 1) was performed not earlier than three months after COVID-19 following the NICE definition of PCS [11]. For the longitudinal analysis, the only inclusion criterion was participation at BL, no additional exclusion criterion was applied. 42 of the initially included 52 individuals agreed to FU assessment and were re-assessed six months after BL. Ten individuals dropped out of the study for different reasons: unspecified reasons (*n* = 4), expectancy of overburdening during cognitive assessment (*n* = 2), no interest in further diagnostics (*n* = 2), long-term stay in a rehabilitation institution (*n* = 1), loss of interest in participation due to symptom improvement (*n* = 1).

### Data assessment and processing

During follow-up (FU) visit, self-reported PCS symptoms were assessed in a structured interview. Besides an extensive neuropsychological test battery that covered the domains of learning and memory, complex attention, executive functions, language, and perceptual-motor function (guided by DSM-5 [33]), cognitive screening tests Mini-Mental State Examination (MMSE [34]) and Montreal Cognitive Assessment (MoCA [35]) were conducted. The domain of social cognition was not covered to reduce test duration. Information on neuropsychological tests and corresponding cognitive domains are listed in Online Resource Table 1. Additionally, symptoms of anxiety and depression (Hospital Anxiety and Depression Scale; HADS [36]), fatigue (Fatigue Severity Scale; FSS [37]), daytime sleepiness (Epworth Sleepiness Scale; ESS [38]), sleep quality (Pittsburgh Sleep Quality Index; PSQI [39]), and health-related quality of life (Short-Form-36 Health Survey Questionnaire; SF-36 [40]) were assessed by self-rating scales. The cut-off for the FSS is defined in the manual as > 4 points on average. Derived from this, we defined a scale cut-off of > 36 points. For the total general health score of the SF-36, we calculated the unweighted mean of the domain-specific values (range 0–100, with lower values indicating worse health ratings).

Based on normative data of the extensive cognitive assessment and the DSM-5 definition of neurocognitive disorder (NCD), individuals were classified as having NCD if at least two test scores indicated a cognitive deficit (i.e., the test result was at least one SD below the mean of the norm). Otherwise, individuals were classified as not having a neurocognitive disorder (noNCD).

In individuals with NCD, cognitive domains were classified as impaired when the result was at least one standard deviation (SD) below the norm’s mean in one test of a cognitive domain.

We created domain-specific cognitive composite scores (DCS) by computing each domain’s unweighted mean of the z-standardized test scores. The DCS for learning and memory, executive functions, and complex attention consisted of five cognitive subscores each, the DCS language consisted of two, and the DCS perceptual-motor function consisted of one subscore. The categorisation of cognitive test scores into cognitive domains was based on theoretical assumptions. Moreover, we created a global cognitive composite score (GCCS) as the unweighted mean of the five DCS.

Additionally, as we focus on cognitive performance changes between BL and FU, we computed difference scores for all DCS and the GCCS by subtracting BL scores from the corresponding FU scores for each individual and then computing the mean of those differences.

### Statistical analyses

The dropout sample (*N* = 10) and FU sample (*N* = 42) were tested for significant differences in cognitive and neuropsychiatric variables at BL. To test for differences in potentially confounding cognitive and neuropsychiatric variables between BL and FU within both the total FU cohort and NCD groups, we performed (a) Welch’s t-test for paired samples to compare sum scores of MMSE, MoCA, PSQI, ESS, HADS anxiety, HADS depression, FSS, the SF-36 total score as well as SF-36 subdomain scores and (b) exact McNemar-tests based on binomial probability testing for paired samples on proportions of subjectively reported fatigue, subjectively reported cognitive impairment as well as scores below/above cut-off scores indicating pathological scores for HADS anxiety, HADS depression, ESS, PSQI and FSS.

To analyze changes in the NCD group distribution (NCD versus noNCD) from BL to FU, we performed the exact McNemar-Test based on binomial probability testing for paired samples.

To test for associations of overall domain impairment distribution and time point of assessment among individuals with NCD, we performed Fisher’s exact test for count data.

Further, we analyzed changes in DCS and the GCCS from BL to FU assessment in the total FU cohort and within NCD subgroups using Welch t-Test for paired samples. Finally, we computed and tested Pearson’s correlation coefficients of the association between the time in days that have passed since infection until FU assessment for both the total FU cohort and the NCD subgroup only.

Statistical analyses were conducted using R (Version 4.3.1). All statistical tests were performed at α = 0.05. Due to the exploratory nature of all the analyses, we did not correct for multiple testing, following the recommendation of Bender & Lange [41].

## Results

### Demographics and clinical variables

The sociodemographic and clinical characteristics of the 42 individuals included are presented in Table 1. There were no significant differences in relevant variables at BL between individuals who were re-assessed at FU and those who dropped out (see Online Resource Table 2).

The number of individuals classified as having NCD at BL was 26 (61.9%) and decreased to 18 (42.86%) at FU. More individuals remitted from NCD than individuals newly developed NCD from BL to FU (*p* = .035). Neither age nor years of education differed between NCD groups at BL or FU (all *p*s ≥ 0.21).

In the total sample, mean health-related quality of life (range 0–100, higher value reflects better health status) increased significantly from *M* = 43.95 up to *M* = *5*4.81 (*t*(41)=-4.41, *p* < .001). Additionally, the score also increased for noNCD (*t*(15) = -2.76, *p* = .015) and NCD groups (*t*(25)=-3.41, *p* = .002) separately, indicating increased health-related quality of life at FU.

**Table 1:** Sociodemographic and clinical variables at baseline (BL) and follow-up (FU) for the total sample and separately for individuals with and without cognitive impairment (NCD; noNCD)

	Baseline		Follow-up		
	*M*	*SD*	*M*	*SD*	
Age	45.69	10.36	46.21	10.29	
noNCD NCD	48.00 44.27	8.63 11.22	48.63 44.73	8.55 11.13	
Days between infection and NPA	243.88	121.74	448.14	126.17	
noNCD NCD	248.38 241.12	141.03 111.14	455.69 443.50	144.55 116.24	
Years of education	15.79	2.28			
noNCD NCD	15.63 15.88	2.22 2.36			
Premorbid IQ	107.44	10.94			
noNCD NCD	108.27 106.96	10.82 11.19			
	*N*	%	*N*	%	*p*
Subjectively reported cognitive complaints	40	95.24	37	88.10	0.375
noNCD NCD	15 25	93.75 96.15	14 23	87.50 88.46	> 0.999 0.500
Subjectively reported fatigue	32	76.19	32	76.19	> 0.999
noNCD NCD	10 22	62.5 84.62	12 20	75.00 76.92	0.688 0.727

*Note*. noNCD (no neurocognitive disorder, *n* = 16) and NCD (neurocognitive disorder, *n* = 26) refer to the classification of individuals based on BL neuropsychological assessment; NPA = Neuropsychological Assessment

**Fig. 1:**
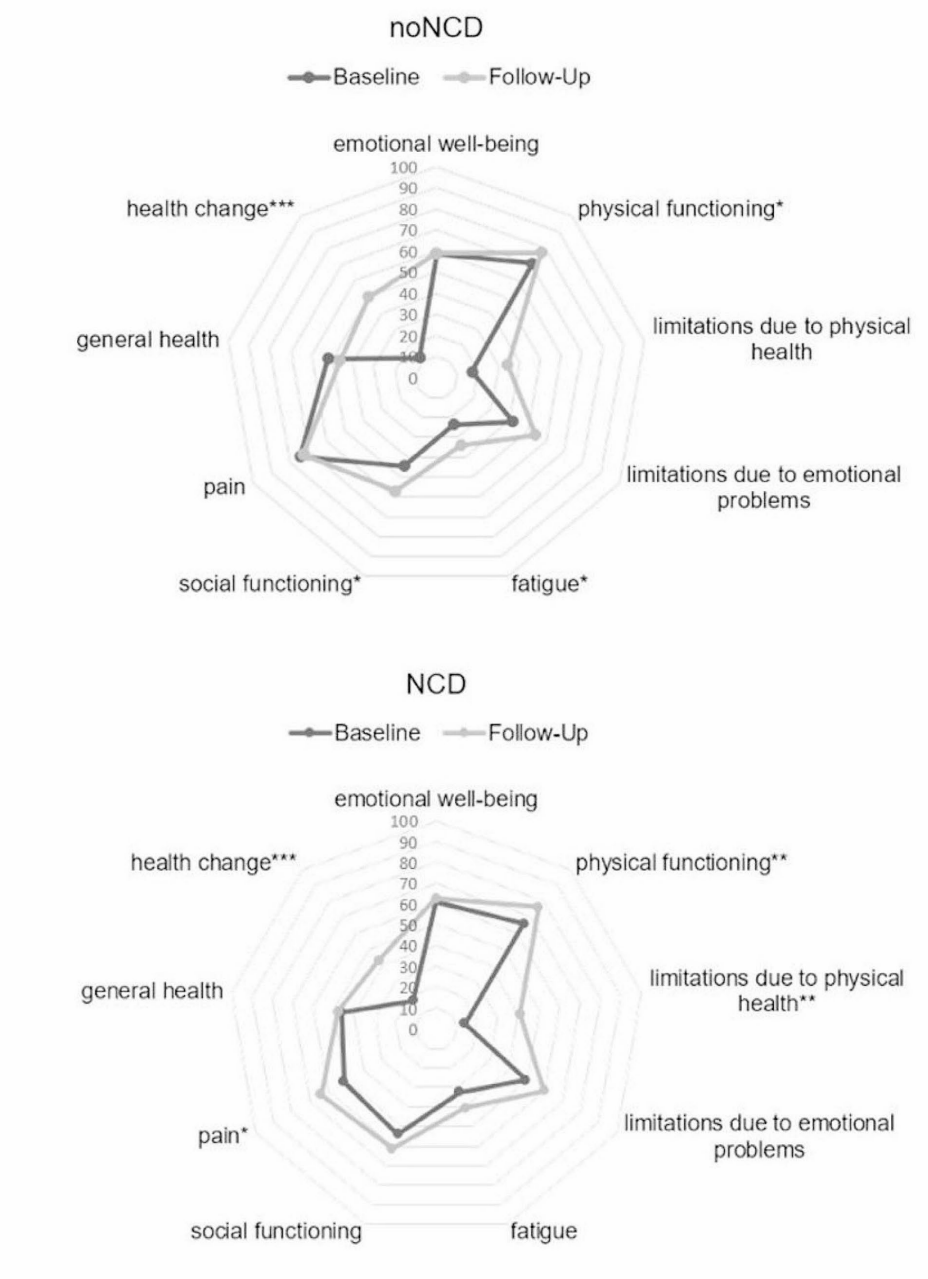
Health-related quality of life measured by SF-36 at BL and FU separately for individuals with noNCD and NCD. *p < .05 **p < .01 ***p < .001

Figure 1 depicts the health-related quality of life at BL and FU separated by subgroups. It shows that when investigating the different subdomains of health-related quality of life more closely (see Fig. 1, Online Resource Table 3), individuals with noNCD at BL improved significantly for physical functioning (*t*(15)=-2.73, *p* = .015), fatigue (*t*(15)=-2.24, *p* = .040), social functioning (*t*(15)=-2.83, *p* = .013), and health change (*t*(15)=-4.24, *p* < .001). For individuals with NCD, physical functioning (*t*(24)=-3.08, *p* = .005), limitations due to physical health (*t*(24)=-3.26, *p* = .003), pain (*t*(25)=-2.66, *p* = .014), and health change (*t*(23)=-3.89, *p* < .001) increased over time. Overall, NCD and noNCD groups showed similar patterns of health-related quality of life subdomains at FU. Both groups’ quality of life was most affected by fatigue. Further, they reported role limitations due to their physical health (e.g., problems at work or other regular daily activities due to physical health) even though limitations of physical functioning (activities like walking, climbing stairs or lifting) were least impaired. Other less impaired health ratings were found for the subdomains pain and social functioning.

**Table 2:** Cognitive screening tests and neuropsychiatric characteristics at BL and FU for the total sample and separately for individuals with and without cognitive impairment (NCD; noNCD)

*N* = 42	Baseline		Follow-up		
Variable (Cut-Off)	*M*	*SD*	*M*	*SD*	*p*
MMSE	29.36	0.93	29.52	0.94	0.376 ^b^
noNCD NCD	29.69 29.15	0.60 1.05	29.44 29.58	1.31 0.64	0.468 ^b^ 0.054 ^b^
MoCA ^d^	26.59	2.83	27.10	2.42	0.218 ^b^
noNCD NCD	27.47 26.08	1.96 3.15	26.5 27.46	2.85 2.08	0.270 ^b^ **.021** ^b^
HADS anxiety	6.76	3.21	6.78	4.25	0.934 ^b^
noNCD NCD	6.38 7.00	3.18 3.26	7.00 6.64	5.10 3.71	0.518 ^b^ 0.562 ^b^
HADS depression	6.55	3.81	6.43	4.73	0.838 ^b^
noNCD NCD	7.13 6.19	4.40 3.44	7.38 5.85	5.52 4.17	0.776 ^b^ 0.660 ^b^
FSS – fatigue	42.69	13.13	38.90	16.32	0.103 ^b^
noNCD NCD	44.44 41.62	13.59 13.00	42.81 36.5	15.58 16.59	0.640 ^b^ 0.105 ^b^
PSQI – sleep quality	8.86	3.64	8.45	4.13	0.365 ^b^
noNCD NCD	8.38 9.15	3.69 3.65	8.31 8.54	4.25 4.14	0.929 ^b^ 0.298 ^b^
ESS – daytime sleepiness	9.71	5.73	9.48	5.01	0.694 ^b^
noNCD NCD	10.63 9.15	5.25 6.03	10.38 8.92	5.34 4.82	0.801 ^b^ 0.770 ^b^

*Note*. noNCD (no neurocognitive disorder, *n* = 16) and NCD (neurocognitive disorder, *n* = 26) refer to the classification of individuals based on baseline neuropsychological assessment

^a^ exact McNemar-test based on binomial probability testing for paired samples

^b^ Welch’s t-test for paired samples

^c^*n* = 39

^d^*n* = 40

^e^*n* = 41

No other significant differences were found for demographic and clinical variables (see Table 1).

Table 2 presents cognitive screening scores and neuropsychiatric characteristics from BL as well as FU. On average, cognitive screening tests showed no evidence of cognitive impairment (MMSE: cut-off < 27; MoCA: cut-off ≤ 25 for impaired cognition) at BL and FU. On an individual level, ten individuals had a MoCA score below the cut-off at FU (BL = 11), while one individual had a MMSE score below the cut-off at FU (BL = 1). Further, mean MoCA and MMSE scores did not improve from BL to FU for the total sample. However, MoCA revealed a significant increase for individuals with NCD over time. On an individual level, ten individuals had a MoCA score below the cut-off at FU (BL = 11), while one individual had a MMSE score below the cut-off at FU (BL = 1).

Similarly, no significant changes were found for neuropsychiatric scores on anxiety, depression, fatigue, sleep quality, and daytime sleepiness between BL and FU. On average, no pathological scores were found for any scale except for fatigue (FAS: cut-off > 36) in the total sample and the subgroups. Sleep quality was poor on average (PSQI: score 6–10 for poor sleep quality, > 10 chronic sleep disturbance), and daytime sleepiness was elevated (ESS: score 8–10 for elevated daytime sleepiness, > 10 for strongly elevated daytime sleepiness).

### Domain composite score (DCS) and global cognition composite score (GCCS)

Individuals with NCD showed impairments in all cognitive domains examined. Descriptively, domain-specific impairments in this group decreased from BL to FU in each domain: Impairments in learning and memory decreased from 69.2 to 55.6%, in executive functions from 61.5 to 44.4%, in complex attention from 50.0 to 38.9%, in language from 42.3 to 33.3%, and in perceptual-motor function from 30.8 to 27.8%. However, Fisher’s exact test did not find an association between domain impairment distribution and assessment time point among individuals with NCD (*p* > .999). The ratio of impairment comparing cognitive domains did not change between BL and FU, i.e., most deficits were found in the learning and memory domain, followed by executive functions, complex attention, language, and perceptual motor functions.

Table 3 compares DCS and GCCS for the total sample and the subgroups (NCD versus noNCD) at BL and FU. When comparing DCS and GCCS between BL and FU in the total FU population and the subgroups using Welch’s t-test for paired samples (see Table 3), mean global cognitive performance of individuals classified as NCD at BL increased significantly for the GCCS (*M* = − 0.38, 95% CI[-0.54,-0.23], *t*(25) = − 5.10, *p* < .001) and explicitly in the mean DCS of learning and memory (*M* = − 0.31, 95% CI[− 0.55,−0.07], *t*(25) = − 2.65, *p* = .014), executive functions (*M* = − 0.38, 95% CI[− 0.57,−0.19], *t*(25) = − 4.07, *p* < .001), and complex attention (*M* = − 0.64, 95% CI[− 0.83,−0.45], *t*(25) = − 6.98, *p* < .001) (Table 3). Additionally, in the domain of complex attention, mean performance of individuals classified as noNCD significantly improved (*M* = − 0.43, 95% CI[− 0.79,−0.07], *t*(15) = − 2.57, *p* = .021) as well as of the total FU cohort (*M* = − 0.56, 95% CI [− 0.89,−0.23], *t*(80.90) = − 3.42, *p* = .001). No significant effects were found for the domains of language and perceptual-motor functions.

While there were significant differences in DCS and GCCS scores, we found no correlation between GCCS and the days that have passed between infection and FU assessment, neither for the total sample (*r* = .05, *t*(40) = 0.32, *p* = .751), nor for the NCD group alone, (*r* = .01, *t(24)* = 0.03, *p* = .975).

**Table 3:** Comparisons of domain-specific composite scores (DCS) and global cognition composite score (GCCS) for the total sample and separately depending on level of cognitive impairment (NCD versus noNCD at BL) for BL and FU

*N* = 42	Baseline		Follow-up		Difference score		
Composite Score	*M*	*SD*	*M*	*SD*	*M*	*SD*	*p*
CS Learning and memory	-0.14	0.75	0.08	0.79	0.22	0.68	0.200
noNCD NCD	0.22 –0.36	0.50 0.80	0.29 − 0.05	0.72 0.82	0.07 0.31	0.80 0.59	0.734 **0.014**
CS Executive functions	0.06	0.67	0.25	0.67	0.18	0.71	0.218
noNCD NCD	0.41 − 0.15	0.46 0.69	0.27 0.23	0.78 0.61	-0.14 0.38	0.91 0.47	0.553 **< 0.001**
CS Complex attention	0.06	0.71	0.62	0.79	0.56	0.56	**0.001**
noNCD NCD	0.49 − 0.21	0.31 0.75	0.92 0.43	0.70 0.80	0.43 0.64	0.67 0.47	**0.021** ** < 0.001**
CS Language	-0.05	0.82	0.11	0.89	0.17	0.77	0.380
noNCD NCD	0.40 − 0.34	0.73 0.76	0.40 − 0.07	0.93 0.84	-0.01 0.27	0.81 0.74	0.968 0.074
CS Perceptual-motor function	-0.07	0.89	0.11	1.00	0.18	1.06	0.382
noNCD NCD	0.32 –0.32	0.69 0.92	0.30 − 0.01	1.03 0.98	-0.02 0.31	1.27 0.92	0.943 0.101
Global Cognition CS	-0.03	0.56	0.23	0.65	0.26	0.55	0.051
noNCD NCD	0.37 − 0.27	0.32 0.54	0.44 0.11	0.68 0.60	0.07 0.38	0.73 0.38	0.721 **< 0.001**

### Conversion rates within neuropsychiatric scales

For neuropsychiatric variables, we observed conversions of both directions, i.e., individuals who converted from being under the cut-off for a certain neuropsychiatric scale at BL to being over the cut-off at FU and vice versa.

**Fig. 2:**
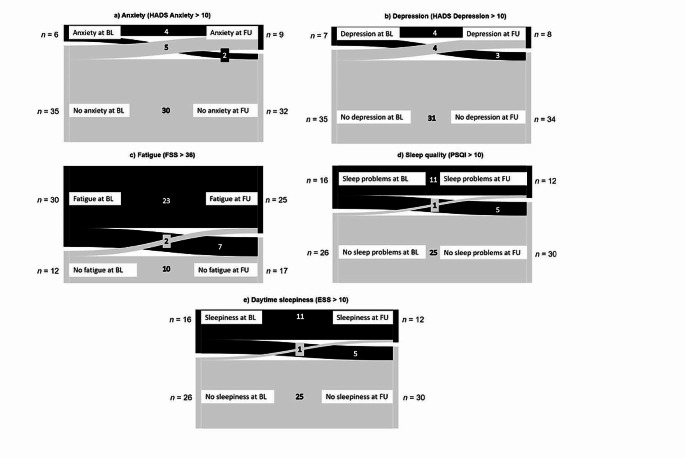
Flow diagrams between BL and FU visits depicting pathological and non-pathological anxiety, depression, fatigue, sleep quality, and daytime sleepiness scores

Figure 2 depicts participant flow of pathological versus non-pathological neuropsychiatric conditions between BL and FU visits. It shows that for anxiety and depression, the total number of individuals, reaching scores above the cut-off, increased descriptively (see Fig. 2a and b). However, McNemar-Tests based on binomial probability testing for paired sampled did not yield significance (*p* = .453 and *p* > .999, respectively), indicating no difference in the probability for the direction of conversion. For fatigue, sleep quality and daytime sleepiness, the total number of individuals showing scores above the cut-off decreased descriptively, while McNemar-Tests did not indicate significance (all *p*s ≥ 0.109).

## Discussion

While a growing number of studies are focusing on cognitive deficits and neuropsychiatric symptoms in individuals with PCS, only a few assess the trajectory of PCS symptoms over time. In this follow-up (FU) study on our prospective monocentric cohort that initially reported subjective cognitive complaints, we applied extensive neuropsychological testing, including five cognitive domains and several neuropsychiatric variables six months after the baseline (BL) assessment in *N* = 42 individuals. We found a significant change in the proportions of neurocognitive disorder (NCD) from BL to FU. For individuals with NCD, cognitive performance (GCCS; MoCA) improved significantly over time, but not for the total sample. Further, we observed significant improvements in the domains of learning and memory as well as executive functions for individuals with NCD, and in the domain of complex attention for the total sample. However, 42.9% of the sample were still classified as NCD at FU (compared to 61.9% at BL). Neuropsychiatric variables like anxiety, depression, fatigue, sleep quality, and daytime sleepiness were stable over time, while on average, the sample reported improved health-related quality of life.

We found that objective cognitive impairment was still present nine months or more after infection in 42.9% of participants, similar to previous studies on PCS [42, 43]. However, we found that global cognitive performance improved significantly from BL to FU for individuals with NCD. Other studies that focused on populations with different acute COVID-19 severity found mixed results regarding the trajectory of cognitive performance in PCS: while Rass et al. [44] found no difference in cognitive performance between three and 12 months after acute infection, Cysique et al. [32] could show a cognitive decline from two to four to 12 months after infection. Our findings most likely support those of Ferrucci et al. [45] who reported cognitive impairment five months after hospital discharge in 63.2% of the sample reduced to 49.1% 12 months later.

Additionally, 88.1% still reported subjective cognitive complaints at FU, compared to 95.2% at BL, indicating no significant difference. Thus far, findings of similar studies reveal discrepancies between subjective cognitive complaints and objective test results. For instance, Schild et al. previously showed that subjective cognitive complaints could be confirmed in no more than half of the sample by objective neuropsychological testing [46], neither in global cognitive performance nor when differentiated by cognitive domains. Other data suggest that subjective cognitive dysfunction is not associated with objective cognitive screening tests and that rates are irrespective of disease severity [47].

Several reasons might contribute to this finding. For example, individuals may have a high premorbid level of cognitive performance so that PCS impairments cannot be objectified by neuropsychological testing at a single time point without a premorbid baseline, and thus do not fulfil the criteria for NCD; impairments may be relatively mild and hence escape objectification; the selected neuropsychological tests may not be sensitive enough to detect cognitive impairment in this group reliably. Results from both BL and FU assessments of this study showed that cognitive screening tests were not as sensitive in detecting cognitive impairment as extensive neuropsychological testing. Further, the neuropsychological profiles of cognitive domains remained similar from BL to FU, with a focus on impairment in the domains of learning and memory, executive functioning, and complex attention. These were also the domains in which individuals with NCD significantly improved the most and were assessed extensively in our study and other studies according to a systematic review of objective test data of cognitive impairment in PCS [10]. Thus, these results could also be interpreted as the need for equally comprehensive assessments within all cognitive domains. However, it is essential to note that applying exhausting assessments could not only be critical for individuals with PCS who experience severe symptoms of fatigue but would also, by chance, lead to more test scores falsely indicating impairment, so that criteria for diagnosing cognitive impairment should be adapted accordingly [48]. Harmonization of neuropsychological assessment could prevent this and increase comparability between studies. A first proposal of internationally harmonized procedures and methods for assessing neurocognitive functions in research and clinical contexts and other health-relevant variables has been published [49]. It is recommended to use inclusive assessment tools for diverse social backgrounds and to revise pre-pandemic norms for neuropsychological diagnostic [50].

Similar to the rates of subjectively reported fatigue and cognitive impairment, psychiatric symptoms, specifically depression, anxiety and fatigue symptoms as well as sleep quality, remained stable between BL and FU. Only health-related quality of life showed significant improvement over time, especially in subdomains physical functioning and health change. These findings support results from other longitudinal studies that reported unchanged or increased rates of neuropsychiatric symptoms over time in PCS [8, 51]. Considering that cognitive performance seems to remit while neuropsychiatric symptoms seem to remain stable, it is unclear if and how cognition and psychiatric symptoms might be associated with each other. Regression analyses that predict cognitive performance and its trajectory from neuropsychiatric symptoms might help uncovering the relationship between cognition and neuropsychiatric symptoms. While this could be seen as an obvious analysis to perform in the context of this study, the ratio of possible predictors to sample size would have led to low statistical power that would have made the results hardly interpretable. Besides traditional regression analyses, other frameworks and methods might be considered in future studies, e.g., network perspectives that reflect the high complexity and likely multifactorial causes of those symptoms and their relationship to each other [52]. Scharfenberg et al. argue that these perspectives are not only able to explain cognitive and neuropsychiatric symptoms in PCS, but similar persisting symptoms observed after other viral infections as well [52].

Accordingly, our results from patients with PCS confirm findings from a systematic review and meta-analysis on SARS and MERS reporting impairments in the domain of memory and of concentration and attention after the acute phase of the disease [28]. Unfortunately, there are not enough data on other cognitive domains to directly compare them with data from patients with PCS. Moreover, health-related quality of life was also significantly reduced after acute SARS infection compared to general population disease [28]. Again, a direct comparison with our data is not possible since the authors focused on selected subscales of the SF-36 and did not have BL data for comparison. However, what we can deduce from these comparisons is that all coronavirus diseases can have a persistent impact on cognition and quality of life.

Our study has several limitations. First, we did not include a control group but compared individuals to pre-pandemic normative data. Thus, initial impairment at BL could have been (partially) caused by circumstances of living in the pandemic rather than due to an infection with COVID-19 [53]. Hence, observed improvement in global cognition could be interpreted in the context of returning to prepandemic life. Our finding that individuals with NCD and individuals with noNCD improved significantly from BL to FU in the domain of attention could support this interpretation. However, this is not a consistent pattern throughout our results and subjective reports of cognitive complaints and stable neuropsychiatric symptoms contradict this interpretation.

Second, the generalization of our findings is limited by the relatively small sample size and demographic characteristics of this German sample. While other studies conducted assessments at specific time points, e.g., starting from infection or hospital discharge, we initially included individuals who already experienced persistent symptoms for at least three months, resulting in a wide range of time between infection and neuropsychological assessments. However, we found no significant association between the time that had passed between infection and FU assessment in this cohort. We therefore do not assume that the trajectory of cognitive impairment due to PCS is tightly associated with the time passed since infection. This mitigates this limitation and suggests that different clusters might exist among individuals with PCS, in which persistent symptoms and their possible remission occur at different rates over time, depending on complex interactions between various factors. Large databases of demographic, neuropsychological and clinical data are needed to identify such clusters using machine learning algorithms (see [54]).

Third, as we only assessed individuals who initially reported subjectively cognitive complaints, prevalence rates of cognitive impairment and psychiatric symptoms should not be generalized to individuals with PCS not reporting subjective deficits. Although the criteria for classifying NCD is based on the DSM-5 NCD criteria, some might argue that the criterion of at least two test scores indicating impairment by being at least one SD below the mean of the norm leads to overestimating NCD in this sample. However, results of BL assessment showed that the NCD groups actually differ in their cognitive performance based on the DCS and GCCS and thus being indeed meaningful in differentiating groups based on cognitive performance.

Moreover, the dropout rate of 19.2% was high. When comparing the FU sample with the dropouts regarding relevant variables at BL (Online Resource Table 2), we found no significant differences, indicating that dropouts were unrelated to systematic differences in initial symptom severity. Only one individual stated that they refused to participate because of subjective cognitive improvement.

Finally, it has to be stressed that all of the analyses presented were of exploratory nature and have to be interpreted accordingly. This limitation is a characteristic of most of the current scientific literature on PCS and not restricted to this specific study. Based on results of these exploratory studies, future studies could be able to define clear hypotheses that then will be investigated in confirmatory hypothesis testing.

A strength of our study is that we extensively assessed cognitive performance in five cognitive domains and several neuropsychiatric symptoms in a well-defined group of individuals with PCS, including the PCS time criterion, in a longitudinal design six months after BL assessments. This approach allowed us to analyze the trajectory of PCS symptoms in a within-subject design, improving the validity of our findings and statistical power. Moreover, since most people with COVID-19 experienced a mild infection [17], explicit knowledge of this specific population about PCS and its trajectory over time is highly relevant.

The current results underline the necessity of developing preventive and therapeutic strategies in PCS and other viral infections [23]. Damiano et al. [23] highlight the necessity to educate health managers and health professionals finding appropriate ways to deal with the topic of cognitive impairment following viral infections by cognitive rehabilitation techniques or therapeutic agents. A fundamental first step is that general practitioners should take cognitive complaints of patients after viral infections seriously, regardless of acute infection severity. Easy and uncomplicated pathways of referral to neuropsychologists should be established to not only diagnose but also to treat cognitive impairments.

Therapeutic strategies should be based on comprehensive (neuro)psychological diagnostic and could include psychotherapeutic treatments to tackle psychiatric symptoms as well as cognitive training interventions for rehabilitating cognitive performance [48, 55, 56]. Meta-analyses show that cognitive trainings are effective in improving cognition in individuals with mild cognitive impairment [57, 58]. These trainings could be used to specifically target cognitive functions that persist to be impaired over time, as well as booster improvements in those that slowly remit. However, they still have to prove to be effective in this specific patient group. There is a number of ongoing trials to test rehabilitation of psychiatric and cognitive symptoms of PCS [59].

Further interdisciplinary research should focus on more extended FU periods in larger samples, including all stages of initial disease severity considering factors that might have negative impact on cognition like sleep disorder, anxiety, depression and fatigue. This procedure is necessary to learn more about trajectories of subjective and objective cognitive impairment and the neuropsychiatric characteristics of PCS and their complex interaction. Additionally, longitudinal intervention studies with regular FU for specific risk groups and symptomatic clusters are the foundation for developing effective and standardized treatment programmes for individuals suffering from PCS [50].

## Electronic supplementary material

Below is the link to the electronic supplementary material.

Supplementary Material 1
