# Supplementary material for: Six-month follow-up of multidomain cognitive impairment in non-hospitalized individuals with post-COVID-19 syndrome
Source: Eur Arch Psychiatry Clin Neurosci. 2024 Jul 24;274(8):1945–57. doi: 10.1007/s00406-024-01863-3 (PMC11579205; doi:10.1007/s00406-024-01863-3)
Supplement: Supplementary file 1 — Supplementary Material 1 [file 406_2024_1863_MOESM1_ESM.docx]

**SUPPLEMENTARY MATERIAL (SM)**

**This file contains Supplementary Material to**

Schild, A.K.*, Scharfenberg, D.*, Regorius, A., Klein, K., Kirchner, L., Goereci, Y., Lülling, J., Meiberth, D., Schweitzer, F., Fink, G.R., Jessen, F., Franke, C., Onur, O.A., Jost, S., Warnke, C.**, Maier, F.** (2024). *Six-month follow-up of multidomain cognitive impairment in non-hospitalized patients with post-COVID-19 syndrome*. European Archives of Psychiatry and Clinical Neuroscience. https://doi.org/10.1007/s00406-024-01863-3

* shared first authorship, Ann-Katrin Schild, Daniel Scharfenberg

** shared last authorship, Clemens Warnke, Franziska Maier

**Corresponding author**:

Dr. Ann-Katrin Schild

Department of Psychiatry, Medical Faculty, University Hospital Cologne AöR, Kerpener Str. 62 50937 Cologne, Germany

Phone: +49 221 478-32298

E-mail: ann-katrin.schild@uk-koeln.de

**TABLE OF CONTENT**

| **SM Item** | **Description/Title** | **Page** |
| --- | --- | --- |
| Supplementary Table 1 | Mean z-scores and standard deviations of the different cognitive tasks based on test-specific normative data in subjects younger or equal to/older than 50 years of age representing the basis for the aggregated domain composite scores | 2 |
| Supplementary Table 2 | Comparison of follow-up sample and dropouts | 4 |
| Supplementary Table 3 | Comparison of SF-36 domains between baseline and follow-Up | 5 |
| References | References used in this file | 7 |

**SUPPLEMENTARY MATERIAL**

**Supplementary Table 1**

*Mean z-scores and standard deviations of the different cognitive tasks based on test-specific normative data in subjects younger or equal to/older than 50 years of age representing the basis for the aggregated domain composite scores*

| Domain composite score | | Cognitive task | | Cognitive measure | | | |
| --- | --- | --- | --- | --- | --- | --- | --- |
|  | Mean (SD) | Total *N* = 42 | Mean (SD)  *N* = 42 | < 50 years  *n* = 29 | Mean (SD) | ≥ 50 years  *n* = 13 | Mean (SD) |
| *Learning & memory* | 0.08 (0.79) | Short term memory | 0.18 (1.06) | WMS digit span forward^a^ | -0.03 (1.11) | WMS digit span forward^a^ | 0.66 (0.77) |
|  |  | Verbal learning | 0.33 (1.15) | VLMT Sum rounds 1-5 ^b^ | 0.44 (1.09) | CERAD wordlist sum rounds 1-3^c^ | 0.08 (1.28) |
|  |  | Verbal recall | 0.03 (1.10) | VLMT round 7 ^b^ | 0.16 (0.96) | CERAD wordlist recall ^c^ | -0.25 (1.36) |
|  |  | Verbal recognition | -0.12 (0.93) | VLMT recognition ^b^ | -0.06 (0.86) | CERAD recognition ^c^ | -0.26 (1.11) |
|  |  | Visual recall | -0.02 (1.27) | WMS-IV visual recall II^d^ | 0.28 (1.14) | CERAD visual recall ^c^ | -0.68 (1.34) |
| *Complex attention* | 0.62 (0.79) | Selective attention | 0.39 (1.13) | TMT-A^e^ | 0.50 (1.08) | TMT-A^e^ | 0.15 (1.26) |
|  |  | Divided attention | 0.70 (0.91) | TMT-B^e^ | 0.70 (0.61) | TMT-B^e^ | 0.71 (1.40) |
|  |  | Attention performance | 0.92 (1.02) | FAIR performance score ^f^ | 1.12 (1.02) | FAIR performance score ^f^ | 0.48 (0.89) |
|  |  | Attention quality | 0.21 (1.11) | FAIR quality score ^f^ | 0.05 (1.04) | FAIR quality score ^f^ | 0.58 (1.22) |
|  |  | Attention continuity | 0.88 (1.04) | FAIR continuity score ^f^ | 1.03 (1.06) | FAIR continuity score ^f^ | 0.54 (0.93) |
| *Executive functions* | 0.25 (0.67) | Processing speed | -0.10 (0.93) | LDST ^g^ | -0.13 (0.97) | LDST ^g^ | -0.03 (0.84) |
|  |  | Working memory | 0.12 (1.07) | WMS span backward^a^ | 0.01 (1.12) | WMS span backward^a^ | 0.36(0.94) |
|  |  | Stroop read | 0.02 (0.83) | Stroop read ^h^ | -0.06 (0.88) | Stroop read ^h^ | 0.21 (0.70) |
|  |  | Stroop name | 0.56 (0.88) | Stroop name ^h^ | 0.52 (0.92) | Stroop name ^h^ | 0.64 (0.81) |
|  |  | Stroop interference | 0.64 (0.75) | Stroop interference ^h^ | 0.57 (0.87) | Stroop interference ^h^ | 0.81 (0.33) |
| *Perc.-motor function* | 0.11 (1.00) | Visual reproduction | 0.11 (1.00) | WMS-IV visual recall I^d^ | 0.31 (0.89) | CERAD constructional praxis ^c^ | -0.34 (1.11) |
| *Language* | 0.11 (0.89) | Phonem. word fluency | 0.10 (1.00) | RWT s-words ^i^ | -0.10 (1.01) | CERAD s-words ^c^ | 0.55 (0.85) |
|  |  | Semantic word fluency | 0.12 (1.16) | RWT animals ^i^ | 0.24 (1.14) | CERAD animals ^c^ | -0.15 (1.21) |

^a^Digit span forward and backward from the Wechsler Memory Scale-Revised (WMS-R) [1]; ^b^ Verbaler Lern- und Merkfähigkeitstest [2]; ^c^ Consortium to Establish a Registry for Alzheimer's Disease (CERAD+) [3, 4]; ^d^ Wechsler Memory Scale IV (WMS-IV) visual recall I and II [5], ^e^ Trail-Making-Test (TMT)-A and -B [6]; ^f^ Frankfurter Aufmerksamkeitsinventar (FAIR-2) [7]; ^g^ Letter Digit Substitution Test (LDST) [8]; ^h^ Farbe-Wort-Interferenz-Test (FWIT) [9], ^i^ one-minute verbal fluency task “s-words” or “animals” [4, 10]; Perc.-motor = Perceptual-motor; Phonem. = Phonematic

**Supplementary Table 2**

*Comparison of follow-up (sample and dropouts)*

|  | Dropouts (*N* = 10) | | Follow-up sample  (*N* = 42) | |  |  |  |
| --- | --- | --- | --- | --- | --- | --- | --- |
| Variable (Cut-Off) | *M* | *SD* | *M* | *SD* | *df* | *t* | *p* |
| age | 49.70 | 10.01 | 45.69 | 10.36 | 13.97 | 1.13 | .277 |
| Years of education | 14.4 | 2.99 | 15.79 | 2.28 | 11.617 | -1.37 | .195 |
| Premorbid IQ | 109.3 | 7.15 | 107.44 | 10.94 | 20.686 | 0.66 | .519 |
| Days between infection and NPA | 241.4 | 116.22 | 243.88 | 121.74 | 14.11 | -0.06 | .952 |
| SF-36 total score | 51.66 | 20.59 | 43.95 | 13.94 | 11.04 | 1.12 | .285 |
| MMSE (< 27) | 29.5 | 0.71 | 29.36 | 0.93 | 17.34 | 0.54 | .598 |
| MoCA (< 26) ^a^ | 27.1 | 3.60 | 36.59 | 2.83 | 11.85 | 0.42 | .681 |
| HADS Anxiety (> 10) | 6.89 | 4.89 | 6.76 | 3.21 | 9.53 | 0.07 | .942 |
| HADS Depression (> 10) | 5.56 | 4.85 | 6.55 | 3.81 | 10.217 | -0.58 | .577 |
| FSS – Fatigue (≥ 36) | 43.10 | 13.61 | 42.69 | 13.13 | 13.29 | 0.09 | .933 ^a^ |
| PSQI – sleep quality (> 10) | 6.67 | 5.36 | 8.86 | 3.64 | 9.64 | -1.17 | .270 |
| ESS – daytime sleepiness (≥ 11) | 8.80 | 6.60 | 9.71 | 5.73 | 12.43 | -0.40 | .693 |

^a^ *n* = 41

**Supplementary Table 3**

*Comparison of SF-36 domains between baseline and follow-up*

|  | Baseline | | Follow-up | |  |
| --- | --- | --- | --- | --- | --- |
| Domain | *M* | *SD* | *M* | *SD* | *p* |
| SF-36 total score – health-related quality of life | 43.95 | 13.94 | 54.81 | 21.86 | < .001 |
| noNCD  NCD | 43.96 43.95 | 16.33 12.59 | 53.89 55.37 | 24.18 20.79 | .015 .002 |
| SF-36 emotional well-being | 60.10 | 16.25 | 61.27 | 21.86 | .772 |
| noNCD  NCD | 58.75 60.92 | 17.99 15.40 | 59.00 62.72 | 24.85 20.12 | .951 .762 |
| SF-36 physical functioning | 67.50 | 23.54 | 76.59 | 24.68 | <.001 |
| noNCD  NCD | 70.63 65.58 | 21.70 24.83 | 77.19 76.20 | 26.33 24.12 | .015 .005 |
| SF-36 limited by physical health | 15.24 | 28.98 | 38.41 | 40.73 | .001 |
| noNCD  NCD | 17.19 14.00 | 29.89 28.94 | 34.38 41.00 | 38.60 42.62 | .085 .003 |
| SF-36 limited by emotional problems | 46.59 | 46.85 | 57.72 | 44.74 | .142 |
| noNCD  NCD | 41.66 49.74 | 47.92 46.87 | 54.17 60.00 | 46.95 44.10 | .252 .332 |
| SF-36 fatigue/energy | 29.02 | 15.34 | 38.05 | 21.91 | .006 |
| noNCD  NCD | 23.75 32.40 | 13.72 15.62 | 34.06 40.60 | 24.51 20.17 | .040 .073 |
| SF-36 social functioning | 50.30 | 24.77 | 59.82 | 27.96 | .004 |
| noNCD  NCD | 44.53 53.85 | 25.81 23.92 | 57.03 61.54 | 31.61 25.96 | .013 .080 |
| SF-36 pain | 60.12 | 29.11 | 67.20 | 28.73 | .046 |
| noNCD  NCD | 74.53 51.25 | 27.96 26.56 | 72.66 63.85 | 29.16 28.52 | .642 .014 |
| SF-36 general health | 48.55 | 19.58 | 47.44 | 22.25 | .805 |
| noNCD  NCD | 52.13 46.35 | 22.82 17.41 | 46.46 48.00 | 23.36 21.98 | .228 .517 |
| SF-36 health change | 15.49 | 25.07 | 45.63 | 32.47 | <.001 |
| noNCD  NCD | 12.50 17.40 | 20.41 27.88 | 50.00 42.71 | 36.51 29.93 | <.001 <.001 |

**References**

1. Elwood RW (1991) The Wechsler Memory Scale—Revised: psychometric characteristics and clinical application. Neuropsychology review 2:179–201. https://doi.org/10.1007/BF01109053

2. Lux S, Helmstaedter C & Elger CE (1999) Normierungsstudie zum Verbalen Lern- und Merkfähigkeitstest (VLMT). Diagnostica 45:205–211. https://doi.org/10.1026//0012-1924.45.4.205

3. Morris JC, Heyman A, Mohs RC, Hughes JP, van Belle G, Fillenbaum G, Mellits ED, Clark C (1989) The Consortium to Establish a Registry for Alzheimer’s Disease (CERAD). Part I. Clinical and neuropsychological assesment of Alzheimer’s disease. Neurology 39:1159–1159. https://doi.org/10.1212/wnl.39.9.1159

4. Schmid NS, Ehrensperger MM, Berres M, Beck IR, Monsch AU (2014) The Extension of the German CERAD Neuropsychological Assessment Battery with Tests Assessing Subcortical, Executive and Frontal Functions Improves Accuracy in Dementia Diagnosis. Dementia and Geriatric Cognitive Disorders Extra 4:322–334. https://doi.org/10.1159/000357774

5. Petermann F, Lepach AC (2012) Wechsler Memory Scale - Fourth Edition, German Edition, 4. Edition. Pearson Assessment, Frankfurt am Main

6. Reitan RM (1958) Validity of the Trail Making Test as an Indicator of Organic Brain Damage. Perceptual and Motor Skills 8:271–276.  https://doi.org/10.2466/PMS.8.7.271-276

7. Moosbrugger H & Oehlschlägel J (2011) Frankfurter Aufmerksamkeits-Inventar 2 (FAIR-2), 2. Edition. Hogrefe, Göttingen

8. van der Elst W, van Boxtel MPJ, van Breukelen GJP, Jolles J (2006) The Letter Digit Substitution Test: Normative Data for 1,858 Healthy Participants Aged 24–81 from the Maastricht Aging Study (MAAS): Influence of Age, Education, and Sex. Journal of Clinical and Experimental Neuropsychology 28:998–1009. https://doi.org/10.1080/13803390591004428

9. Bäumler G (1985) Farbe-Wort-Interferenztest (FWIT) nach J.R. Stroop. Hogrefe, Göttingen

10. Aschenbrenner, S, Tucha, O & Lange KW (2000) Regensburger Wortflüssigkeits-Test (RWT), 1. Edition. Hogrefe, Göttingen
